# Health Service Utilization among Syrian Refugees with Chronic Health Conditions in Jordan

**DOI:** 10.1371/journal.pone.0150088

**Published:** 2016-04-13

**Authors:** Shannon Doocy, Emily Lyles, Laila Akhu-Zaheya, Arwa Oweis, Nada Al Ward, Ann Burton

**Affiliations:** 1 Johns Hopkins Bloomberg School of Public Health, Baltimore, MD, United States of America; 2 Jordan University of Science and Technology, Irbid, Jordan; 3 World Health Organization, Amman, Jordan; 4 United Nations High Commissioner for Refugees, Amman, Jordan; National Institute for Viral Disease Control and Prevention, CDC, China, CHINA

## Abstract

**Introduction:**

The influx of Syrian refugees into Jordan presents an immense burden to the Jordanian health system, particularly in treating chronic health conditions. This study was undertaken to assess utilization of health services for chronic health conditions among Syrian refugees in non-camp settings.

**Methods:**

A survey of Syrian refugees in Jordan was undertaken in June 2014 to characterize health seeking behaviors and issues related to accessing care for hypertension, diabetes, cardiovascular diseases, chronic respiratory diseases, and arthritis. A cluster design with probability proportional to size sampling was used to attain a nationally representative sample of 1550 non-camp Syrian refugee households.

**Results:**

Of 1363 cases with a chronic health condition diagnosis, 84.7% had received care in Jordan. Public facilities faced a heavy burden serving over half (53.9%) of care-seekers; the remainder received care in the private (29.6%) and NGO/charity (16.6%) sectors. Individuals with non-communicable diseases (NCDs) in the central region of Jordan and with arthritis had the lowest rates of care-seeking when compared to other regions and conditions. Overall, 31.6% of care-seekers had an out-of-pocket payment for the most recent care-seeking event which averaged 18.8 USD (median = 0 USD), excluding cost of medications.

**Discussion:**

Forced displacement presents major challenges to those with NCDs, which have the potential to seriously impact both the quality of life and life expectancy amongst refugees. NCD patterns among Syrian refugees indicate the importance of continuing support to public sector services in Jordan to adequately meet expanding needs and ensure appropriate prevention and control of priority NCDs.

## Introduction

With more than four million registered refugees and another 7.6 million internally displaced, Syrians are the world’s largest conflict-affected population [1,2]. Jordan has over 629,000 registered Syrian refugees, most of whom (>80%) are settled among Jordanian communities, not in camps [3]. This is reflective of broader global trends, where refugees are increasingly coming from middle income countries and reside in non-camp settings [4]. The growing displacement of older populations from middle income countries brings a higher burden of chronic health conditions and unique sets of needs which pose new challenges for humanitarian agencies and host country governments [5,6]. In response to this shift, humanitarian agencies have adapted existing assistance modalities to focus on integration of refugees into host country health systems [7]. The changing profile of displaced populations requires a longer-term focus on ensuring continuity of care, access to medications, and adequate secondary and tertiary services. However, despite significant investments in health infrastructure and health systems strengthening activities, the resulting strain of refugee populations on host countries remains immense.

Syrian refugees registered with UNHCR in Jordan were able to access primary, secondary, and some tertiary healthcare free of charge at Ministry of Health facilities before late 2014 and out-of-pocket payments were not required for many services. Due to the burgeoning refugee population and the high cost of care, currently refugees are required to pay out-of-pocket at the same rate as uninsured Jordanians. Although this is still highly subsidized, the costs of both accessing and providing uninterrupted care for chronic health conditions can be considerable and may be a barrier to care, especially given recent declines in humanitarian assistance [8]. Inadequate routine care for chronic conditions can lead to complications requiring sophisticated treatments and preventable adverse health outcomes [5]. This study was undertaken to assess access and utilization of health services for chronic health conditions among Syrian refugees in non-camp settings in Jordan.

## Methods

A survey of Syrian refugees in Jordan was conducted in June 2014 to characterize health seeking behaviors and understand issues related to accessing services. A cluster survey design with probability proportional to size sampling was used to attain a nationally representative sample of Syrian refugees outside of camps. Sample size was determined based on a conservative prevalence estimate of 50% for key indicators; calculations assumed 80% power and a design effect of 2.0. The minimum identified size (n = 900 households) was increased to 1500 households to account for non-response and provide additional power for detection of significant differences of >10% between sub-national regions.

**Fig 1:**
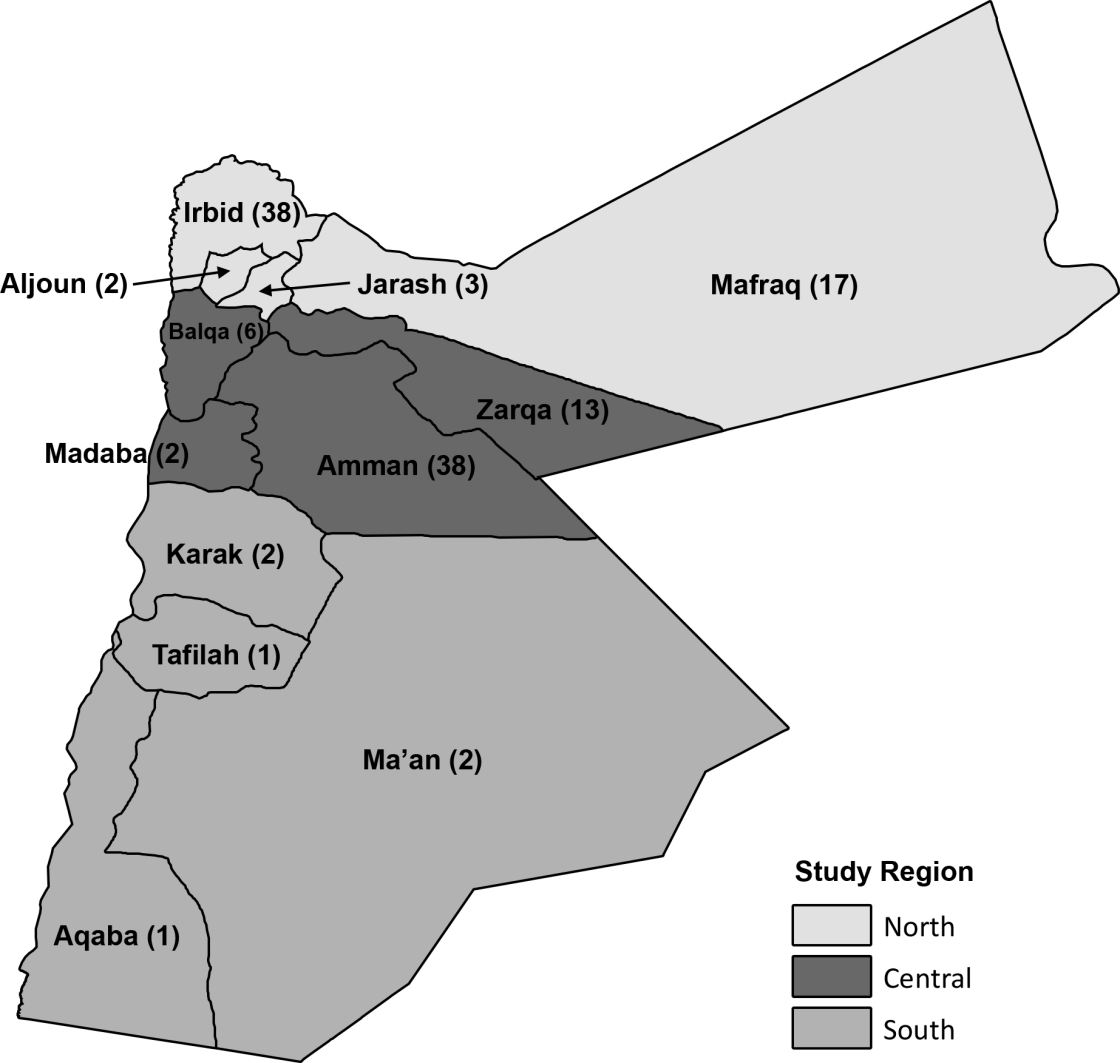
Distribution of Study Clusters.

A 125 cluster x 12 household design was chosen because of the relative ease of accessing numerous locations and because smaller clusters were logistically preferable. UNHCR registration data were used to assign clusters at the sub-district level; it was assumed that registered and non-registered refugees had similar settlement patterns. The final cluster assignment included 38 clusters (30%) in Amman, 38 clusters (30%) in Irbid, and 49 clusters (40%) distributed proportionately in the remaining governorates (Fig 1). Governorates were allocated into three regions for analysis: (i) North (Aljoun, Mafraq, Irbid, and Jarash governorates), (ii) Central (Balqa, Amman, Zarqa, and Madaba governorates), and (iii) South (Aqabah, Karak, Tafilah, and Ma’an governorates). The UNHCR Amman Office randomly selected five households listed as living in that cluster’s assigned sub-district from their registration database. Households were called and the first household residing in the specified sub-district that agreed to meet was used as the starting point for the cluster. The study team conducted an abbreviated interview (which was excluded to minimize bias towards registered refugees) and enquired about Syrian households in the vicinity, which were subsequently interviewed. This referral method was used until the cluster was complete. Respondents were most often household heads or caretakers of children, and they answered questions on behalf of the households; where possible other household members were engaged to improve quality of responses for selected pertinent questions. Household members were defined as people who share a dwelling space and meals, regardless of biological relation; short-term visitors, staying less than one month, were excluded. At the conclusion of each interview, a referral to the nearest Syrian household was requested; the referral process was used until twelve interviews were completed. Only Syrian households arriving in 2011 or after were eligible to participate; however, none of the households approached arrived in Jordan before 2011.

The questionnaire was developed following discussions between partners; by consensus, the focus was on health service utilization, access and barriers to care, children’s health, and chronic health conditions. Respondents were asked about the five chronic conditions perceived to be most common among the Syrian refugee population: hypertension, cardiovascular diseases (including heart failure, angina, arrhythmias [irregular heartbeats], a previous heart attack, or previous stroke), diabetes, chronic respiratory diseases (including asthma, chronic bronchitis, emphysema, and chronic obstructive pulmonary disease), and arthritis [9,10]. Cases were identified through self-reported diagnosis of the condition from a health professional. If more than one household member of any age had a particular condition, one was randomly selected and a series of questions on health service utilization and the most recent visit for the chronic health condition was asked about that household member.

The questionnaire was translated to Arabic, translated back to English to check that the meaning had not changed, and pilot tested multiple times. Interviewers, who were nursing students and faculty, underwent two days of classroom training followed by one day of field training. To protect the anonymity of respondents, no unique identifiers were recorded and verbal consent was obtained. Verbal consent was obtained rather than written consent because of high illiteracy rates and for confidentiality protection, where the consent form would be the only document linking the subjects to participation in the survey as no other unique identifiers were collected. Oral consent was documented in the electronic data record. All ethics committees and Internal Review Boards approved this method of verbal consent. Interviews lasted between 30–60 minutes and data were collected on tablets using the Magpi platform (DataDyne LLC, Washington, DC). Data were analyzed using Stata 13 (College Station, TX) and Tableau Desktop (Seattle, WA). Standard descriptive statistics and methods for comparison of means and proportions and accompanying 95% confidence intervals were used; the Stata ‘*svy’* command was used to account for the cluster design and adjusted standard errors for design effects. Logistic regression was utilized to determine associations between background characteristics and care-seeking variables of interest. Variables with statistical significance in univariate logistic regression (p<0.01) were incorporated into multiple logistic regression models. Manual forward selection was performed to add variables that were not significant in univariate analysis but strengthened the overall model according to an adjusted Wald test; this process allowed each model to contain only variables significantly associated with the outcome. All cost figures are presented in USD at an exchange rate of 0.71 JD/US$ [11].

The study was approved by the Jordanian Ministry of Health (MoH) and ethics committees at the World Health Organization, Jordan University of Science and Technology, and Johns Hopkins School of Public Health.

## Results

A total of 1634 households were approached; of these 2.9% (n = 47) were not at home, 0.8% (n = 14) were previously interviewed under the same study, and 1.4% (n = 23) declined. The final sample included 1550 households with 9580 members, which equates to a 94.7% response rate. The average household size was 6.2 (CI: 6.0–6.4; range 1–20) and 95% (CI: 93.6–96.1) of households were currently registered or waiting for an appointment with UNHCR. Approximately half (50.3%, CI: 47.3–53.4) of households had member(s) previously diagnosed with one of the five chronic conditions of focus. The final sample included 1363 cases from 780 households that were interviewed about care seeking for their condition.

### Health Facility Utilization

**Fig 2:**
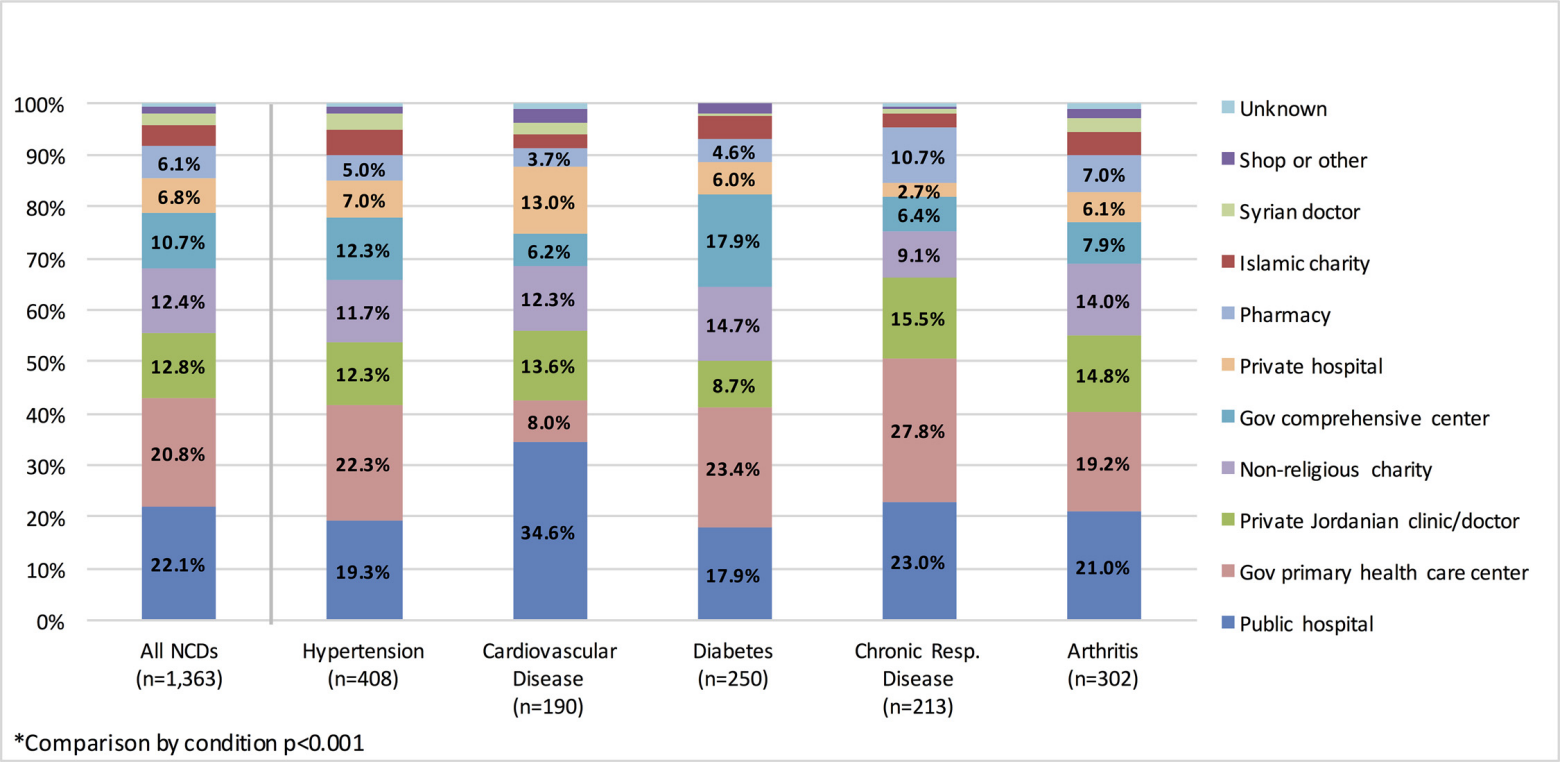
Facility Type Utilized for Most Recent Chronic Disease Care in Jordan by Condition*.

The care-seeking rate, defined as the proportion that sought care last time they perceived it was needed, for chronic health conditions was 84.7% (CI: 81.6–87.3). Health facility utilization by condition and sector are summarized in Table 1 and Fig 2. Care-seeking rates were between 85.3–87.8% for all conditions except arthritis, which was significantly lower at 75.8% (p<0.001). Despite the concentration of infrastructure in Amman, refugees in the central governorates of Jordan had significantly lower care-seeking rates than in other regions (p = 0.004). When examined by sector, more than half of visits for a chronic health condition occurred in public facilities (53.9%, CI: 49.2–58.5); private facilities accounted for 29.6% (CI: 25.7–33.7) of visits followed by charity/NGO facilities (16.6%, CI: 13.1–20.8) and pharmacies (6.1%, CI: 4.4–8.2). Of all conditions, chronic respiratory disease cases had the highest utilization rates of public and private sector facilities, at 57.5% and 30.7%, respectively. Compared to the other surveyed chronic conditions, diabetes cases utilized charity/NGO facilities at the highest rate (19.3%) and were least likely to seek care in the private sector (21.6%).

**Table 1:** Most Recent Medical Care Received in Jordan for Chronic Health Conditions.

	N	Sought Care for a Chronic Condition ^a^	Sector in Which Most Recent Care Was Sought ^b^		
			Public	Private	Charity/NGO
		% (95% CI)	% (95% CI)	% (95% CI)	% (95% CI)
All NCDs	1,363	84.7 (81.6,87.3)	53.9 (49.2,58.5)	29.6 (25.7,33.7)	16.6 (13.1,20.8)
HT	408	87.7 (83.8,90.8)	54.2 (48.1,60.2)	28.9 (23.9,34.5)	16.9 (12.4,22.5)
Cardio. Disease	190	85.3 (79.2,89.8)	49.4 (40.9,57.9)	35.6 (28.5,43.5)	15.0 (10.0,22.0)
Diabetes	250	87.2 (81.8,91.2)	59.2 (51.8,66.2)	21.6 (16.0,28.3)	19.3 (14.3,25.5)
Chronic Resp. Disease	213	87.8 (83.1,91.3)	57.5 (50.1,64.6)	30.6 (24.1,38.1)	11.8 (7.7,17.8)
Arthritis	302	75.8 (69.8,80.9)	48.5 (41.2,55.7)	33.0 (26.6,40.1)	18.5 (13.6,24.7)
*p-value for comparison by condition*		---	0.024		

Non-communicable diseases (NCDs); Hypertension (HT); 95% Confidence Interval (95 CI); Non-governmental organization (NGO).

^a^ As a percent of total number of index cases reporting diagnosis of condition.

^b^ As a percent of those seeking care in Jordan for condition.

### Predictors of Care Seeking

Results of univariate and multivariate logistic regression analyses for predictors of care-seeking for chronic health conditions are presented in Table 2 and S1 Table. Household head education status was significantly related to care-seeking in univariate analysis; however, after controlling for additional covariates of interest using multinomial logistic regression, the relationship was no longer significant. Geographic region of residence in Jordan and the specific health condition reported were significantly associated with care seeking in both univariate and multivariate regression. With regard to condition, individuals with cardiovascular diseases and diabetes had 3.83 (CI: 1.20–12.20 and 1.21–12.16, respectively) times higher odds of care-seeking than those with hypertension in the adjusted model; while no significant differences in care-seeking rates were observed for other conditions. Additionally, odds of care-seeking for households in the central governorates of Jordan were 0.35 (CI: 0.16–0.80) times those in northern Jordan in the adjusted model; no significant differences in care-seeking rates were observed for households in southern Jordan.

**Table 2:** Care-Seeking for Chronic Health Conditions (n = 1,290)^a^.

	Sample Characteristics	Adjusted Odds of Seeking Care ^b^
	% (95% CI)	OR (95% CI)
**Registered with UNHCR**	94.8 (92.6,96.4)	
**Household Head Educational Attainment (Highest Level Completed)**		
None	17.8 (11.2,27.1)	Reference
Primary	27.3 (19.7,36.4)	0.26 (0.03,2.18)
Preparatory	30.6 (21.3,41.8)	0.33 (0.04,2.89)
Secondary or higher	24.4 (17.1,33.6)	0.19 '(0.03,1.33)
**Socioeconomic Quartile (based on monthly expenditures)**		
Bottom	19.1 (15.9,22.8)	---
2nd	22.9 (19.7,26.4)	---
3rd	27.0 (23.3,31.0)	---
Top	31.0 (26.4,36.0)	---
**Crowding (5+ / sleeping room)**	13.2 (10.7,16.3)	---
**Year of Arrival in Jordan**	** **	** **
2011–2012	43.9 (39.3,48.6)	---
2013–2014	56.1 (51.4,60.7)	---
**Region of Residence**		
North	50.6 (41.1,60.0)	Reference
Central	45.1 (35.9,54.6)	*0*.*35 (0*.*16*,*0*.*80)*
South	4.3 (1.8,9.8)	1.31 (0.24,7.27)
**Chronic Condition**		
Hypertension	29.9 (28.2,31.8)	Reference
Cardiovascular disease	13.9 (12.4,15.7)	*3*.*83 (1*.*20*,*12*.*20)*
Diabetes	18.3 (16.9,19.9)	*3*.*83 (1*.*21*,*12*.*16)*
Chronic Respiratory Disease	15.6 (13.7,17.7)	2.86 (0.83,9.84)
Arthritis	22.2 (20.2,24.2)	0.77 (0.35,1.70)

^a^ "Care-seeking" defined as having sought care last time it was needed.

^b^ Italic indicates statistically significant (p < 0.05) findings.

Many characteristics of care-seeking significantly differed by the sector in which care for a chronic health condition was sought and are presented in S2 Table. A greater proportion of those seeking care in the NGO/charity sector (98.4%, CI: 93.2–99.6) were members of households registered with UNHCR compared to those seeking care in the private (92.3%, CI: 87.1,95.6) and public sectors (95.1%, CI: 92.6–96.9) (p = 0.05). Household head education status was significantly lower among individuals seeking care in the NGO/charity sector followed by the public and private sectors (p = 0.01). Geographically, individuals seeking care in the public and private sectors were spread evenly across the regions, whereas care-seekers in the NGO/charity sector resided mostly in the northern governorates (71.6%, CI: 57.8–82.3, p<0.001). A significantly larger proportion of individuals seeking care in the NGO/charity (22.1%) and public sectors (20.9%) were doing so for diabetes compared to those in the private sector (13.9%). Conversely, a smaller proportion of those seeking care in the NGO/charity sector did so for chronic respiratory diseases (11.6%) compared to the private (16.8%) and public sectors (17.3%). A similar proportion of individuals seeking care in the NGO/charity and private sectors (22.1%) were seeking arthritis care, significantly higher than among public sector care-seekers (17.8%). A higher proportion of care-seekers in the private sector were cardiovascular diseases care-seekers (16.8%) than in public (12.8%) and NGO/charity sectors (12.6%).

### Spending on Health Services for Chronic Health Conditions

Cost of care-seeking for chronic conditions was measured for the most recent visit and are presented in Table 3. Out-of-pocket payments for the consultation, including diagnostic and laboratory tests were included; however, payments for medication or those made on the patient’s behalf by the United Nations or another organization were excluded. Overall, 31.6% (CI: 27.7–35.8) of care-seekers reported an out-of-pocket consultation payment. The average out-of-pocket consultation payment was 18.8 USD (median = 0 USD); among only those who paid for care, the average increased to 59.2 USD (median = 14 USD). These costs account for approximately 3% and 9%, respectively, of reported monthly household expenditures and 6% and 18% of reported monthly household income, relatively high proportions for one care visit for conditions requiring continuous care.

**Table 3:** Out-of-Pocket Expenditures for Most Recent Chronic Disease Care Among Syrian Refugees in Jordan^a^.

			Cost of Consultation (USD)			
		Paid Provider for Consultation ^b^	Overall		Among Cases that Paid for Care	
	N	% (95% CI)	Median	Mean (95% CI)	Median	Mean (95% CI)
All NCDs	1,363	31.6 (27.7,35.8)	0	18.8 (5.9,31.6)	14	59.2 (19.2,99.4)
HT	408	30.7 (25.8,36.1)	0	18.3 (0,41.9)	14	59.6 (0,136.1)
Cardio. Disease	190	32.1 (24.5,40.8)	0	35.4 (0,86.4)	21	110.3 (0,270.6)
Diabetes	250	26.1 (19.8,33.7)	0	28.3 (0,66.7)	14	108.6 (0,253.5)
Chronic Resp. Disease	213	32.6 (26.2,39.8)	0	6.2 (4.4,8.0)	14	18.9 (14.8,23.1)
Arthritis	302	37.1 (30.7,44.0)	0	8.7 (4.7,12.8)	14	23.5 (13.5,33.6)
*p-value for comparison by condition*		0.146		0.453		0.386

95% Confidence Interval (95 CI); Non-communicable Diseases (NCDs); Hypertension (HT).

^a^ All cost figures are presented in U.S. Dollars.

^b^ As a percent of those seeking care in Jordan for condition.

**Fig 3:**
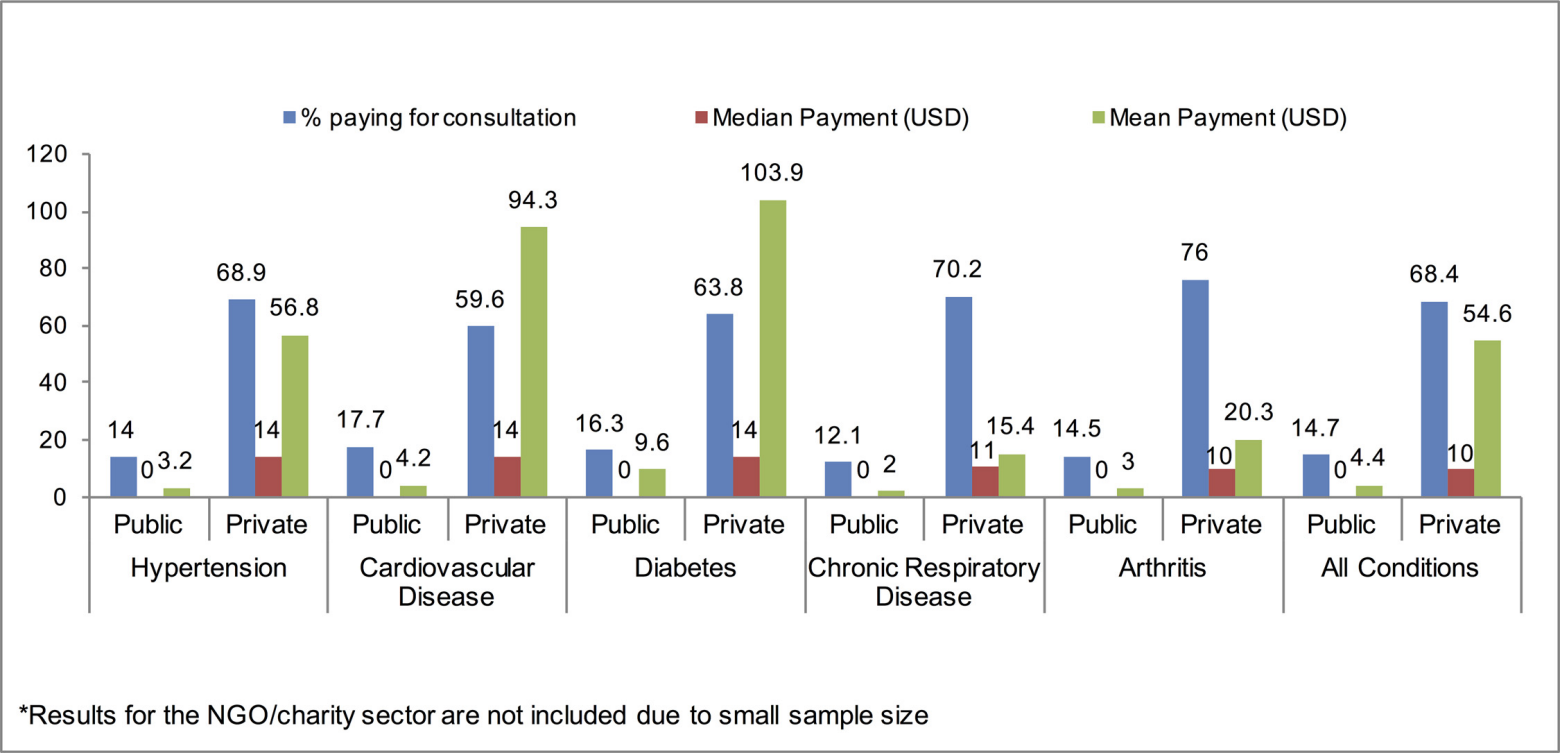
Payments for NCD Consultations in the Public and Private Sectors.

Significant regional differences in the proportion of patients with out-of-pocket payments were observed (p = 0.01) with the highest percentage paying in the central governorates of Jordan (37.8%, CI: 31.4–44.8) and the lowest percentage paying in the northern governorates (26.8%, CI: 22.2–31.9). No significant differences in out-of-pocket payment amounts were observed between regions (p = 0.81); however, the highest mean out-of-pocket costs were observed for diabetes and cardiovascular disease care. While these conditions are chronic in nature, they may have acute exacerbations resulting in high costs to manage and treat severe outcomes. While this may not be the case for many cases reporting payments, the presence of such outliers may have influenced the overall average cost of care for these conditions. Out-of-pocket expenditures by condition and sector where most recent care was received are summarized in Fig 3 and S3 Table. Spending on consultation fees was not significantly different among the five chronic health conditions both by the proportion of patients with an out-of-pocket consultation payment (p = 0.15) and in the amount of payment (p = 0.28). Out-of-pocket payments by sector and condition are summarized in Fig 3. The proportion with payments was significantly higher in the private sector (68.4% private, 14.7% public, 19.5% NGO/charity; p<0.001) and the difference in average payment amount was marginally significant by sector with the highest average payment in private sector (mean 54.6 USD, median 10 USD) compared to public sector (mean 4.4 USD, median 0 USD) and the NGO/charity sector (mean 1.7 USD, median 0 USD) (p = 0.06).

## Discussion

The study demonstrates a high burden of NCDs amongst the displaced Syrian population in Jordan. Using the last time care was needed as an indication, the ability to access care is a good testament to the Jordanian MoH’s generous support to Syrian refugees. Differences in care-seeking by condition may be explained by the nature of the condition and indicate areas of potential intervention. In particular, the lower rate of care-seeking among arthritis cases may be explained by more patients self-managing their condition with over the counter medications and not finding formal health visits necessary. Unlike many chronic conditions, self-management of arthritis has been shown to sustain health benefits with minimal costs [12]. Another explanation for lower care-seeking for arthritis may be reduced mobility making it more difficult for patients to travel to health facilities. The higher utilization rates of public sector facilities may indicate that they are preferred by care-seekers due to their wider presence and coverage of all geographic areas in the Kingdom, their reasonable and affordable cost, and possibly the satisfaction of users with the quality of the service. With the crisis well into its fifth year and displacement protracted, this burden entails substantial costs to UNHCR and other providers as well as increased utilization of Jordanian referral hospitals by refugees.

Disease patterns indicate the importance of continuing support to the public care sector and Jordanian public health services to adequately meet expanding needs. Although cancers were not specifically queried in the survey, the burden is likely to be heavy, particularly on the tertiary care system. In 2010, non-communicable diseases (NCDs) were estimated to account for 46% of all deaths in Syria and mean body mass index and mean fasting blood sugar were increasing in both males and females [13]. With 59% of Syrian males smoking (and 23% of females), consuming a national per capita 1205 cigarettes per year, the health consequences will place a heavy burden on the tertiary health facilities of Jordan [14]. It had previously been estimated that lung cancer is the second most common cause of cancer deaths in Syria [15]. The pattern observed in this study of refugees not seeking care because of costs, not understanding their disease, and being poorly compliant with treatment, increases the probability of more expensive inpatient and specialist care in future years. Very little attention was given to NCD prevention and health promotion in Syria prior to the conflict, making it more difficult to begin to address these in a displaced population [13]. Investing now in more aggressive health promotion could more than pay for itself in future savings from hospital care avoided or postponed.

One important limitation to household surveys is that quality of care cannot be evaluated. Assessments of quality care would be useful for informing health systems strengthening efforts. For example, episodes of shortages or stock-outs of regularly required medicines or adjunct supplies such as spacers, glucose monitoring equipment in insulin requiring diabetes, and peak flowmeters would be useful in determining the frequency of these problems and in designing stock monitoring and supply chain strengthening strategies to address them. Additionally, other measures of access such as the number of visits in the preceding six months and the proportion of refugees whose condition is controlled would be useful to compare against the quality of care and the population’s needs. Previous assessments have demonstrated that care was compromised for displaced Syrians in Jordan with two-thirds of participants in the study reporting that their condition had worsened since leaving Syria. The key barriers cited in the assessment were inability to obtain regular medications due to MoH shortages and out-of-pocket costs for medications [16]. While such results were not exactly aligned with findings from this study owing to the high rate of care-seeking, it does echo reasons cited by those not seeking care in this study and suggests that cost is the principal barrier to care-seeking. At the time of the survey, Syrian refugees in Jordan were entitled to free access to primary and secondary care in Ministry of Health facilities; however, costs were not covered in private facilities, nor for medications outside of the approved essential medicines list. For patients with chronic conditions, cost barriers may still be substantial depending upon where care is sought and which medicines are needed. The policy of free access to public health services has since changed and, effective November 2014, non-camp Syrian refugees access public health care at a subsidized rate equivalent to that paid by uninsured Jordanians. The implications of the increase cost burden to refugees as a result of this change in policy is likely to hinder care-seeking and continuity of care for chronic conditions [17].

Forced displacement presents major challenges to those with NCDs both during flight as well as in the country of asylum. Many common direct (e.g. cardio and peripheral vascular disease, microvascular disease, etc.) and secondary effects (e.g. acute care such as injury care) of inadequately controlled NCDs can often be delayed with continuous adherence to treatment, including prescribed medication. Conversely, in the absence of adequate control of these diseases, such effects are more likely to result in complications that often require costly specialized care. NCDs usually require regular medication and monitoring to ensure stable control. Three common results of displacement have immense implications for health outcomes refugee populations: 1) treatment interruptions due to inability to access medicines with attendant consequences of unstable disease and acute and chronic complications; 2) poor disease monitoring due to inadequate follow up and disruption in home monitoring; and 3) deterioration in lifestyle risk factors such as exercise, smoking, nutrition, stress, and psychosocial effects due to lack of control over living circumstances and experience of traumatic events. The continuing challenges in sufficiently addressing NCDs have the potential to seriously impact both quality of life and life expectancy amongst refugees.

Current guidance on NCDs in emergencies is not adequate [18]. One of six strategic objectives published in UNHCR’s 2014 Global Strategy for Public Health is to “*facilitate access to integrated prevention and control of NCDs including mental health services*” [19]. The focus of this strategy is targeting risk factors for NCDs and promoting an integrated approach to management of NCDs at the primary health care level. In order to be effective, NCD care needs to be integrated throughout all stages of the refugee cycle including in contingency planning. Lessons can be learned from the management of chronic communicable diseases such as malaria, tuberculosis, and HIV in displaced settings.

The Syrian refugee crisis provides an opportunity to document effective interventions and approaches to ensure access to quality multidisciplinary NCD care amongst refugees. In the Middle East, specialists provide much of the care for NCDs with little involvement of the primary care provider. This model may not meet the needs of displaced populations as it can result in a fragmented approach, higher cost services, and a possible reduction in access to care among refugees owing to the fact that specialists tend to be concentrated in large urban centers. Furthermore, the crucial role of nurses and health educators in supporting NCD care is not fully realized. Jordan has a well-developed health system but refugees may need additional support to ensure appropriate prevention and control of priority NCDs.

## Supporting Information

S1 Data Dataset including variables used in analysis of health system utilization for chronic health conditions among Syrian refugees in Jordan. (XLS)

S1 Table Household Characteristics by Care-Seeking for Chronic Health Conditions. (XLSX)

S2 Table Care-Seeking for Chronic Health Conditions by Sector (n = 1,290). (XLSX)

S3 Table Out-of-Pocket Consultation Payment for Most Recent Chronic Disease Care among Syrian Refugees in Jordan by Sector*. (XLSX)
